# Some Thoughts and Facts about the Superstitions of Medicine and Mental Influences as Curative Agents

**Published:** 1886-02

**Authors:** J. S. Todd

**Affiliations:** Atlanta, Ga.


THE ATLANTA MEDICAL AND SURGICAL JOURNAL.

VOL. II. FEBRUARY, 1886. NO. 12.

### Original Communications.

### SOME THOUGHTS AND FACTS ABOUT THE SUPERSTITIONS OF MEDICINE AND MENTAL INFLUENCES AS CURATIVE AGENTS.

BY J. S. TODD, M. D., ATLANTA, GA.

READ BEFORE THE ATLANTA SOCIETY OF MEDICINE DECEMBER, 1885.

Superstition is the natural product of all ignorant, yet thinking minds. The recognition of supernatural power is common to the human family wherever found and under any and all circumstances. Wherever an effect is seen the cause is looked for, and if not recognized among the patent, tangible operations of nature, the imagination plumes its wings, and, soaring above the earth, finds the sought-for origin in the limitless domain of the unreal, the speculative and the supernatural. Let mystery but spring up and a moment brings forth its brood of superstitions. Disease, hidden in its origin, silent in its march, invisible in its hiding-place, and but the forerunner of the great mystery of mysteries is the fountain-head from which spring the superstitions that have darkened the earth.

It is not strange, then, that man, in his early history, before the light of science had shed its rays upon the darkened world—when every calamity was attributed to the wrath of the gods—should come to look upon the physician, he who essayed to peep into the secrets of Omnipotence and arrest the decree of fate, as a supernatural being.

Æsculapius had temples which rivaled in magnificence the mighty Jove himself, and was served by priests who burnt incense and sacrificed on his altars. Toxaris, a Greek medical man of far-famed erudition, who was said to have arrested the plague in a supernatural way, had erected to him by the grateful Athenians an altar on which a white horse was sacrificed every year.

The union, for ages in the same office of priest and physician, did much to give to medicine and medical men reverence, amounting to worship, and shrouded it in a cloud of mystery, which is still unlifted, for medicine has been, and continues to be, an art so conjectural that our astonishment at the anxiety with which empires have been sought after is much diminished.

Many, very many of the superstitions connected with the early history of medicine prevail now, as they did in former times, with this exeeption: then they were universally taught, received and practiced by the learned; now, as a general rule, only the ignorant give them credence, and the large number of the latter class, owing to the superstitions of the negro population of the South, invests the subject with additional importance.

"The blunders of the weak are short-lived, but a false theory struck in the mint of genius, often deceives the learned and passes current through the world."

Dr. Isaac Letsome, one of England's greatest physicians in his day, an ardent disciple of the antiphlogistic treatment of disease, was much worried to find over his sign, which read I. Letsome, the following couplet, placed there by some wag:

"When sick folks do to me apply,
  I purges, vomits, bleeds and sweats 'um;
If after that they choose to die,
  I Letsome."

It has been affirmed, and probably with truth, that during the bleeding era of medicine, the lancet numbered more victims than the sword. Dr. Rush, the grand high priest of medicine in America a little over a hundred years ago, was founder of the first, and now probably the best, school of medicine on the continent. The Doctor was also a minister of the gospel. Gorgeously framed in the museum of the University of Pennsylvania is a prescription he wrote on the fly-leaf of a prayer book in answer to a summons to see a sick girl. In pencil are these words: "Let the wench be blooded. I will see her after the service."

It is not strange, then, that darkness and error should reign so long, when we remember that such men as Pythagoras, Paracelsus, Galen, and even Roger Bacon, in their almost total ignorance of the fearful and wonderful construction of man, this last delicate handiwork of the Creator, believed in alchemy, astrology, charms and amulets.

Astrology, or the science of the stars, gave rise to the study of astronomy. Galen held that a man who was ignorant of astrology was unfit to practice medicine; he was a common cheat and no better than a murderer. The horoscope of every person of importance was taken with special care.

"The moon, that wandering ghost
Which walks in penance nightly,
And sheds so sad a light,
Although it shines so brightly."

has turned the brains of many men. Hence the word lunatic. Bulwer's Ione says:

" I watched its face when a child,
Until it turned my brain ;
And now I often weep to think
'Twill ne'er be right again."

An eclipse of this luminary has always been regarded by the ignorant as an omen full of evil.

Not only the tides and vegetable substances were under the control of pale luna, but all animals born when she presented her stern-visaged profile to our gaze were irritable, weak and short-lived. On the contrary, when her broad and rollicking, red-faced majesty was seen in the east, just after old Sol had retired behind the western hills away, such creatures as she then gazed upon for the first time were in life to be merry, prosperous, vigorous and long-lived.

Our homœopathic and disowned brethren, according to one of their late journals, accord to moonlight gathered during the full, in thin water and shaken until it is lunatic, specific virtues in the cure of certain affections. I can but admire this weapon of the follower of Hahnemann, whose teachings are in theory and practice all moonshine. But be it far from me not to acknowledge that Hahnemann did much for our calling. In fact, there is no system, professional or empirical, from which the philosophical mind fails to cull something that is practical, and he did demonstrate that Dame Nature was an ally worth courting.

But I had rather trust to charms and amulets than his pellets; infinitesimal doses fail to make the impression or work the cures that have been ascribed to the former as agents in medicine and surgery. One of the most intelligent patients I ever had, a terrible sufferer from cramps in the lower limbs, after my remedies were unavailing, was cured by simply tying a flannel cord above the knees. The cramps returned when this was left off, but a second recourse to the same expedient was as successful as the first. The amount of testimony which I have procured as to the efficacy of a pan of cold water under the bed of a person afflicted with night sweats is perfectly overwhelming.

That disease is cured by faith and prayer is so well attested it would be foolhardy in me to contradict.

Over the old houses in Edinburgh there are still remains of talismanic characters which the superstitions of early ages caused to be engraved on their fronts. Our modern man who is superstitious—and who has not a touch of it?—wards off witches and evil spirits by placing over his doorway a horse-shoe, which is certainly cheaper, if not so ornamental.

Which is the more superstitious, the man of old who prayed to St. Anthony (the saint who had rheumatism under his charge), and wrote at the wane of the moon mysterious words on a golden plate wrapped with the sinews of a crane, to be worn near the part affected, or the average American, who, during the summer, carries an Irish potato in his pocket and clothes himself during the winter in red flannel ?

The belief that east winds are forerunners of epidemics, and that they portend evil, is an error deeply grounded in the minds of the people and the medical fraternity. Those religiously inclined probably think that because an east wind brought locusts into Egypt, therefore on its wings can come nothing that is good, not stopping to think that Palestine is east of the Nile. As all lore originally came from the East, the professional men doubtless base their theory on Hindoo medical ideas, for, according to one of their theories, the body is composed of one hundred thousand parts, in which are comprised seventeen thousand vessels, each of which has seven different channels and in which are ten species of wind. Diseases arise from the irregular directions of those winds, and as external air, which enters the lungs in the act of respiration, is the source of all winds, the best preventive of diseases consists in not breathing too quickly. After the air has been purified in its long journey across the Atlantic, it is strange, aye, cruel and senseless, in the citizens of Portland, New York, Norfolk, Savannah or Charleston to dread it or give it a bad report, for an east wind is as much dreaded by the inhabitants of the Pacific coast as along the Atlantic seaboard. If an east wind is deleterious in Savannah, what is the cause of it ? It comes from the sea laden with the chlorides and heavily impregnated with moisture, I almost hear some one say. In Spain the only wind which blows over dry land is from the east, and yet the people say it is an unhealthy one. The north and south winds in France—one bracing, the other a gentle zephyr—are borne on old ocean's bosom. Nevertheless, popular prejudice heaps calumnies on the east wind, laden with the fragrance of Rheinish vineyards, pure from Swiss mountains, balmy from Italy's waving corn-fields.

Accident has caused many discoveries in science and superstition has indirectly led to others. Sir Kenelm Digby's magic powder, that was applied, not to the wound, but to the weapon which inflicted it, gave rise to a rational treatment of surgical injuries; for in course of time sensible men saw that it was the rest and withholding of irritating applications which worked the cure.

St. John, the evangelist, in describing the celestial city, the new Jerusalem, says, in substance, that through the midst of it ran a river of water, clear as crystal, and on either bank grew twelve manner of trees whose leaves were for the healing of the nations. The only tree on this earth comparable to these heavenly ones is the cinchona, from which we procure quinine. The history of its introduction teaches that when carried to Europe, as it was by the Jesuits, "that learned bigotry condemned what unlettered savages had discovered, and one religious body zealously spurned a priceless boon that had been introduced by another whose form of faith they derided." Nor did it overcome "the sneers of the learned or the hate of bigotry until an English quack succeeded in curing a man of high rank, and then fashion broke down the prejudices which reason could not remove."

The metallic tractors of one Perkins created no little stir in their day, but Dr. Hogarth showed that wooden tractors painted in the same style were equally efficacious as remedial agents. It was the effect on the imagination and the belief of the patient that did the good. For it is the confidence of the unblushing charlatan and the faith of the patient that cures. "Imagination," says Lord Bacon, "is next akin to a miracle, a working faith." The old English name of scrofula was king's evil. Hundreds of people were cured of this disease by a touch of the royal fingers. These are historical facts. Von Helmoldt affirmed that he had discovered a metal of which, when made into a ring and worn, not only the pain attendant on hemorrhoids would vanish soon, but that they, be they external or internal, would dry up and disappear in a few days. His rings had a large sale, and wrought many cures. But commend me to the modern, who carries a buck-eye in his pocket for this disease; it is *vegetable—purely a vegetable*—and cannot *ex necessitata rei* be poisonous!

Dr. J. M. Johnson, of this city, well known to many of my listeners, and when I say well-known, I mean favorably and lovingly remembered, not only as a man of science, but a prince among gentlemen, whose sands of life are nearly run out, but, like the sun, seems greatest at his setting, tells me that he "has known a large number of intelligent people to raise black cats for their blood, to be used as a cure for erysipelas."

Electricity, while it has its army of advocates, has never, under my observation, cured a single disease. I have tried to believe in it, but find it is recommended principally for nervous diseases, such as are greatly influenced by the mind. I have just finished reading a book on electricity. In it is chronicled a case of obstinate chills and fever that, resisting all treatment, was finally cured by electricity, the precaution being taken to give the liquor hammondi three times a day in conjunction. I had a curiosity to see what the liquor hammondi was, and in a foot-note I found that it contained strychnine, quinine, iron and phosphorus. How could I avoid being reminded of the brave fellow, who took to the loft when the bear came, and there remained till his wife killed it, and after the smoke of battle had died away, he patted her on the back and said: "Ain't we brave, Nancy?"

It would be a useless, tiresome and profitless waste of time to dwell further on the superstitions that have enveloped medicine and that still control, to a great extent, the action of drugs upon the human body, and hang like a nightmare around the very science itself; for to enumerate them would require a "thousand tongues, a throat of brass and adamantine lungs."

The voudooed or tricked American citizen of African descent was reared seeing around him those who believed that the air was peopled with invisible forms; that the moanings of the wind were requiems to the dead; that strange birds hovered around the hut of him who was soon to commence that journey from whence there was no return; the shadows at twilight were "hands which you could not see, but beckoned him away," the sudden breaking of silence in a solitude "were voices which said he must not stay." To the uneducated mind that accepts the doctrine of the immortality of the soul, the life beyond the grave, the belief in the supernatural, would come to be a doctrine readily accepted; for if faith has wafted the spirits of the dead to the further shore of gloomy Styx, it needs but a short and rapid flight of the imagination to bring it back to the haunts it knew when embodied in the flesh, especially if there were a motive for its return in the way of unsettled scores.

But indeed this strange and weird fancy does not find its slaves alone among the ignorant and unlettered. Nor is it confined to any station in life, or any age, but finds its development as well in the nineteenth century in the person of Ichabod Crane, as, with staring eye-balls and chattering jaws, he belabors the ribs of old Gun Powder, scours the valley of Sleepy Hollow, while hard behind him rides the headless Hessian ; as in the hardy king of Scotland, who, amidst the hosts of his Highland chieftains, trembles at the ghost of murdered Banquo.

Indeed, it is said there is not a tongue on earth that is not rich in legends of the dealings of the spirit-world with this wide-awake, real and sun-lit world of ours. In the face of science, the teachings of religion, the dictates of reason, the scorpion-lash of ridicule held ever threatening up, who can say he has not nor will ever feel the promptings of this strange fear of the unreal? I mean not here this evening, with this bright array of flesh and blood, but I would have you answer the query when alone, in the hush of darkness, the witching time of night when graveyards yawn, and shadows creep forth from among the darkness of burial places. In such a place and among such surroundings let the question be answered. How impotent is reason in the presence of the supernatural! I care not when or where the struggle comes, nor upon whom the test is made. Let it be the philosopher, whose well-balanced mind has weighed all things, and whose judgment tells him that nothing but that which is real can exist. Let it be the dauntless mariner, whose foot has trodden every shore, whose eyes have looked upon all the waters of the earth, whose hardy courage has known no faltering, though the storm-king rode upon the wings of the wind, and the angry sea opened wide its countless mouths. Let it be the hero of an hundred battle-fields, who, amidst shot and shell and blood of conflict, laughed at death and knew not how to fear. Let it be the man who, sustained by a heaven-born courage, walks amidst the air of pestilence unflinchingly in his rounds of duty as he ministers to the wants of dying and suffering humanity. Let him, let them all, who have faced death fearlessly in all its forms, but come face to face, as they dream, with a spectre from the world beyond the grave, and every hair will stand on end, the coward blood rush back to its source, and reason desert her throne.

I do not wish to depreciate the value of drugs as remedial agents. They hold an important place in therapeutics, from which they can never be dislodged. They are not, however, the sole or chief reliance of the physician. They are auxiliary to other or more potent forces, whose subtle influence, unperceived and unrecognized by the illiterate, are often decisive of the issues of life and death.

Every physician is aware of the immense influence the mind sways over the body, and when wise, he utilizes this to his own renown and the patient's good. The patient's surroundings, his clothing, his food, his thoughts, misdirected too often by almanac literation and ingeniously gotton up remedies, are all each and every one of them agents which we are forced in practice to accept as means of cure. The neglect by too many of us of paying attention to these four all-important agencies has led to the almost universal belief that we are limited, or limit ourselves to drugs alone as agents against disease. In answer to the question, "What was your treatment?" most persons, medical and non-medical, expect only a catalogue of the drugs administered.

> " Cure her of that :
> Canst thou not minister to a mind diseased :
> Pluck from the memory a rooted sorrow ;
> Raze out the written trouble of the brain ;
> And, with some sweet, oblivious antidote,
> Cleanse the stuffed bosom of that perilous stuff,
> Which weighs upon the heart ?"

is a question asked by Macbeth of the physician, which must still be answered, "No, not by drugs."

"When we take a retrospective glance at the condition of medicine in former times, and reflect upon the amount of ignorance, credulity and superstition which prevailed, we cannot fail to be struck with the immense improvement that has taken place in comparatively modern periods, and must be encouraged in the hope that as the physical and moral sciences pursue their onward progress, and as the means of observation and experiment are augmented and facilitated, our own noble science may attain a pitch of perfection of which at the present day we can form no adequate conception, shedding light where all is now obscurity, and tending to dispel doubt and difficulty wherever existent." "Let them be terrible in constant resolution," to ennoble, elevate and advance our science. Three-score years and ten are allotted to men to live. That the average human life should be seventy years is our mission. Then, with this grand motto engraven on our banners, "We dare do all that may become men: who dare do more is none:" let us continue the strife against disease and premature death.

